# Cervical ultrasonography has no additional value over negative ^18^F-FDG PET/CT scans for diagnosing cervical lymph node metastases in patients with oesophageal cancer

**DOI:** 10.1007/s00330-017-5136-x

**Published:** 2017-12-07

**Authors:** Lucas Goense, Jihane Meziani, Peter S. N. van Rossum, Frank J. Wessels, Marnix G. E. H. Lam, Richard van Hillegersberg, Jelle P. Ruurda

**Affiliations:** 1 Department of Surgery, University Medical Center Utrecht, Heidelberglaan 100, 3584CX Utrecht, The Netherlands; 2 Department of Radiation Oncology, University Medical Center Utrecht, Utrecht, The Netherlands; 3 Department of Radiology, University Medical Center Utrecht, Utrecht, The Netherlands; 4 Department of Nuclear Medicine, University Medical Center Utrecht, Utrecht, The Netherlands

**Keywords:** Oesophageal cancer, Cervical ultrasonography, ^18^F-FDG positron emission tomography/computed tomography, Cancer staging, Cervical lymph node metastases

## Abstract

**Objectives:**

To investigate the additional value of cervical ultrasonography over ^18^F-FDG PET/CT for diagnosing cervical lymph node metastases in patients with newly diagnosed oesophageal cancer.

**Methods:**

Between January 2013 and January 2016, 163 patients with newly diagnosed oesophageal cancer underwent both cervical ultrasonography and ^18^F-FDG PET/CT at a tertiary referral centre in the Netherlands. Retrospective clinical data analysis was performed to assess the diagnostic value of cervical ultrasonography and ^18^F-FDG PET/CT for the detection of cervical lymph node metastases. Fine needle aspiration or clinical follow-up was used as reference standard.

**Results:**

The overall incidence of patients with cervical lymph node metastases was 14%. The sensitivity of ^18^F-FDG PET/CT to detect cervical lymph node metastases was 82% (95% CI 59–94%) and specificity was 91% (95% CI 85–95%). The sensitivity and specificity of cervical ultrasonography were 73% (95% CI 50–88%) and 84% (95% CI 77–90%), respectively. In patients with a negative ^18^F-FDG PET/CT, 12 of 133 (9%) patients had suspicious nodes on cervical ultrasonography. In all these 12 patients the nodes were confirmed benign.

**Conclusions:**

Cervical ultrasonography has no additional diagnostic value to a negative integrated ^18^F-FDG PET/CT for the detection of cervical lymph node metastases in patients with newly diagnosed oesophageal cancer.

***Key Points*:**

• *Cervical ultrasonography has no value over PET/CT in evaluating cervical node metastases.*

• *PET/CT provides greater diagnostic confidence compared to cervical ultrasonography.*

• *Cervical ultrasonography during standard diagnostic work-up may be considered unnecessary.*

• *Cervical lesions on PET/CT require cytopathological confirmation by FNA.*

## Introduction

Oesophageal cancer is the eighth most prevalent cancer, and the sixth most common cause of cancer-related death worldwide [1]. Surgical resection of the oesophagus with en bloc lymphadenectomy remains the cornerstone treatment with curative intent for patients with non-metastatic oesophageal cancer [2, 3]. Currently, a multimodal treatment approach is increasingly applied since many studies have shown a survival benefit of neoadjuvant chemo(radio)therapy over surgery alone for patients with resectable oesophageal cancer [4–6].

Accurate staging of oesophageal cancer is essential to select patients that are eligible for treatment with curative intent, and to identify patients with distant metastases to prevent a non-curative surgical procedure. Currently recommended staging techniques include endoscopic ultrasound (EUS) with fine needle aspiration (FNA), ^18^F-fluorodeoxyglucose (^18^F-FDG) positron emission tomography (PET) with integrated diagnostic computed tomography (CT) of the neck, chest and abdomen, and cervical ultrasonography with FNA [7]. In some guidelines the use of cervical ultrasonography is recommended since this is considered an effective and accurate approach to assess cervical lymph node involvement [7, 8]. However, the introduction of integrated ^18^F-FDG PET/CT scanning has improved the accuracy of cancer staging by providing both anatomical and metabolic information [9]. Therefore, the additional role of cervical ultrasonography for the detection of cervical lymph node metastases in the current era of routine diagnostic ^18^F-FDG PET/CT imaging may be limited.

Accordingly, the aim of this study was to investigate the additional value of cervical ultrasonography over ^18^F-FDG PET/CT for diagnosing cervical lymph node metastases in patients with newly diagnosed oesophageal cancer.

## Methods

Institutional review board approval was obtained, and requirement for written informed consent was waived for this cohort study. In this study all patients referred to our tertiary referral centre with newly diagnosed oesophageal cancer, between January 2013 and January 2016, were identified from a prospectively collected database. Within this cohort, patients who were evaluated with both integrated ^18^F-FDG PET/CT and cervical ultrasonography were included. The two investigations of interest were performed in random order. Cervical lymph nodes with suspected metastasis on the basis of cervical ultrasonography and/or ^18^F-FDG PET/CT underwent FNA and cytopathological examination. Clinical follow-up data from all patients were collected to identify cervical metastases that were potentially undetected during clinical staging. Therewith, the composite reference standard of the current study included either positive cytopathology of the aspirated material from a suspicious cervical lymph node during initial staging or the occurrence of a new cervical lymph node metastasis that was detected within 12 months of clinical follow-up after initial staging and proven malignant with cytology. Cervical lymph node metastases were grouped into levels corresponding to upper, middle and lower jugular nodes (levels II, III, IV, respectively), and posterior triangle nodes (level V).

After completion of staging, all patients with a clinical stage T1N1–3 or T2–4aN0–3, with no evidence of distant or pathologically confirmed cervical metastases, were scheduled for oesophagectomy with two-field lymphadenectomy.

### Cervical ultrasonography with FNA

Ultrasonography of the cervical and supraclavicular region was performed by experienced radiologists, using a 12.5- to 17.5-MHz linear array transducer (Philips Medical Systems, Best, the Netherlands). Cervical lymph nodes with a short axis diameter of at least 5 mm (at least 7 mm for high jugular/level II nodes), or nodes less than 5 mm with a prominent appearance (round shape, loss of fatty hilum, [focal] low echogenicity or an eccentric mass) and grouped nodes with a short axis diameter of at least 3 mm were considered suggestive of metastasis and cytologically examined after FNA.

### Integrated ^18^F-fluorodeoxyglucose PET/CT

Patients had to stay sober for at least 6 h before injection of ^18^F-FDG, and blood glucose levels were measured to check for potential hyperglycaemia. The administered activity of intravenously administered ^18^F-FDG was 2.0 MBq/kg. Approximately 60 min after administration of ^18^F-FDG, PET and CT imaging were performed from neck to abdomen in all patients using a PET/CT system (mCT, Siemens, Erlangen, Germany). Before PET acquisition, a diagnostic quality iodine contrast-enhanced CT was performed using the following settings: 120 kV, 20 mA, 0.5 s tube rotation time, pitch of 1.0 and 3.0 mm slice width. PET was performed using 3-dimensional acquisition, an axial field of view of 216 mm and a scanning time of 3 min/bed position. ^18^F-FDG PET/CT data were reconstructed using iterative ordered subset expectation maximization for 21 subsets and four iterations (Gaussian filter). All ^18^F-FDG PET/CT images were visually interpreted by experienced nuclear medicine physicians. Cervical nodes were considered suspicious on PET/CT if they had increased non-physiological ^18^F-FDG uptake based on visual interpretation without size constraints. Additional FNA was performed in case of suspicious lymph nodes.

### Follow-up

The duration of clinical follow-up was calculated from the date of last diagnostic work-up (^18^F-FDG PET/CT or cervical ultrasonography) until identification of cervical lymph node metastases, last visit at the outpatient clinic or death. The median follow-up of all eligible patients included in this study was 11 months (interquartile range 6–15 months). The time of follow-up of patients without lymph node metastases that were still alive during follow-up was at least 12 months.

### Statistical analysis

Sensitivity (SE), specificity (SP), positive predictive value (PPV) and negative predictive value (NPV) of ^18^F-FDG PET/CT and cervical ultrasonography for the detection of cervical lymph node metastases were calculated. Sensitivity and specificity were compared using McNemar’s test, and PPV and NPV using the Chi-square test, between ^18^F-FDG PET/CT and cervical ultrasonography, respectively. The additional value of cervical ultrasonography to ^18^F-FDG PET/CT was determined by calculating the proportion of patients with a positive cervical ultrasonography that was confirmed malignant, in the group of patients with a negative ^18^F-FDG PET/CT scan. Differences in diagnostic performance were explored using sensitivity analyses including distant cervical lymph node metastasis (level II), regional cervical lymph node metastasis (level III, IV, V) and squamous cell tumours.

## Results

**Fig. 1:**
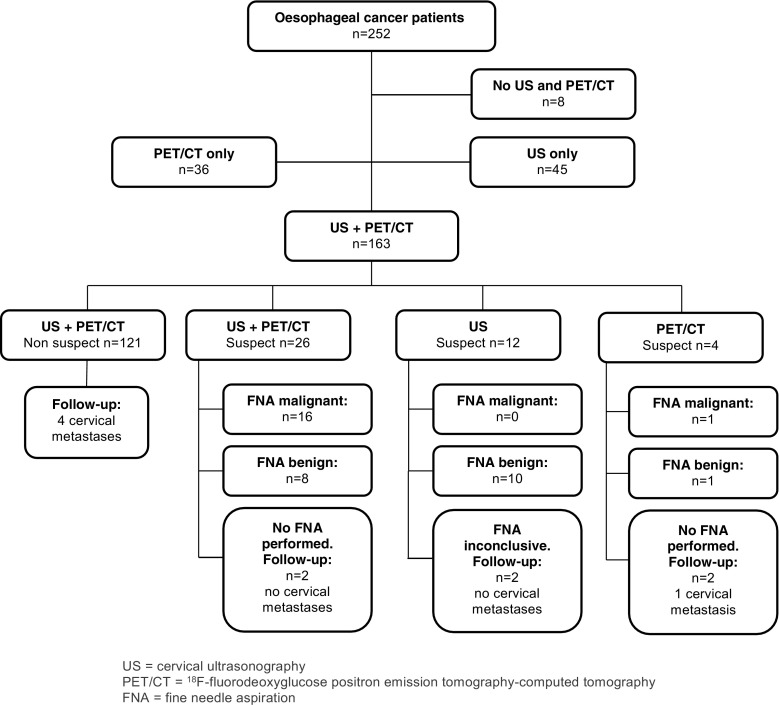
Flowchart of patients (*n* = 163) with primary oesophageal cancer who underwent ^18^F-FDG PET/CT and cervical ultrasonography between January 2014 and January 2016. US cervical ultrasonography, PET/CT ^18^F-fluorodeoxyglucose positron emission tomography–computed tomography, FNA fine needle aspiration

**Fig. 2:**
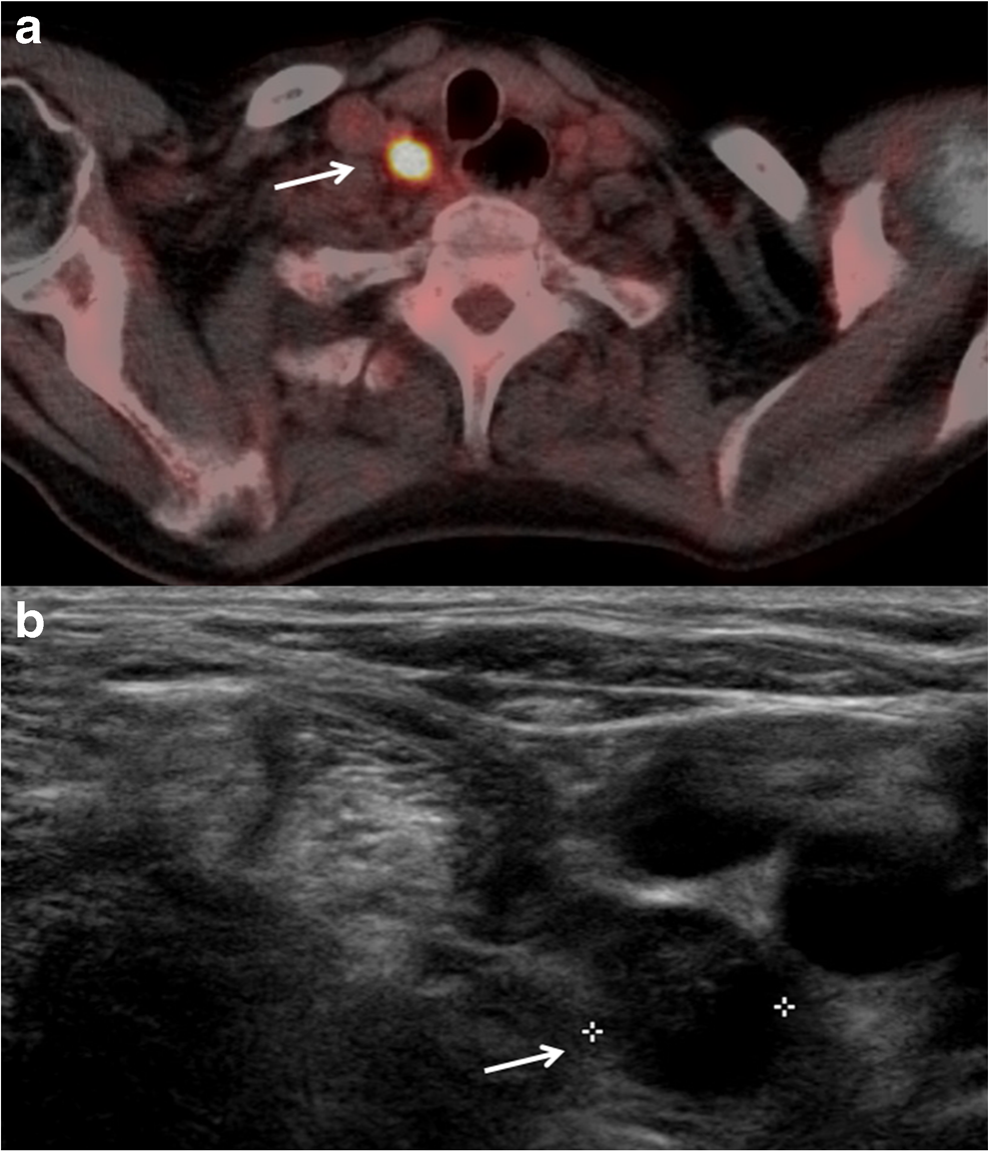
**a** Fused axial PET/CT image demonstrating a 10-mm FDG-avid cervical lymph node metastasis in station IV (right) during initial staging in a patient with a primary oesophageal squamous cell tumour (arrow). **b** Cervical ultrasonography image in the same patient, demonstrating a 10-mm cervical round-shaped lymph node metastasis in level IV (right). The cervical lymph node was confirmed malignant with FNA (arrow)

Between January 2013 and January 2016 252 patients with newly diagnosed oesophageal cancer were referred to our tertiary referral centre. Of these patients, 89 were excluded because ^18^F-FDG PET/CT (*n* = 45) or cervical ultrasonography (*n* = 36) was not performed, or both cervical ultrasonography and ^18^F-FDG PET/CT were not performed (*n* = 8). The main reasons to refrain from cervical ultrasonography of the neck was previous imaging showing distant metastases. In case a ^18^F-FDG PET/CT was not performed, this was mainly due to the gradual introduction of this imaging modality for this indication in our hospital in the first year of the study period. These patients underwent a CT scan only. A total of 163 patients underwent both cervical ultrasonography and ^18^F-FDG PET/CT imaging, and were included in the current analysis. Figures 1 and 2 shows the flowchart of patient selection, and Table 1 demonstrates the patient and treatment-related characteristics. The overall incidence of patients with cervical lymph node metastases was 14% (22/163). Of the 22 cervical lymph node metastasis, 2 (9%), 4 (18%), 15 (68%) and 1 (5%) were located in levels II, III, IV and V, respectively.

**Table 1:** Baseline characteristics

Characteristic	*N* (%)
Male gender	114 (70)
Age (years)^a^	67.2 (±8.6)
Tumour location	
Proximal oesophagus	21 (13)
Middle oesophagus	45 (28)
Distal oesophagus	74 (45)
GEJ/cardia	23 (14)
Histology	
Adenocarcinoma	94 (58)
Squamous cell carcinoma	66 (40)
Other	3 (2)
Clinical T-stage	
T1	16 (10)
T2	25 (15)
T3	102 (63)
T4	18 (11)
Tx	2 (1)
Clinical N-stage	
N0	28 (17)
N1	59 (36)
N2	42 (26)
N3	34 (21)
Clinical M-stage	
M0	143 (88)
M1	20 (12)

*GEJ* gastro-oesophageal junction

^a^Expressed as mean ± standard deviation

### Cervical ultrasonography and ^18^F-FDG PET/CT: non-suspect nodes

Cervical ultrasonography and ^18^F-FDG PET/CT were both negative for cervical lymph node metastases in 121 out of 163 (74%) of the patients, and therefore no FNA was performed. During clinical follow-up, four of these 121 patients developed cervical lymph node metastases, which were confirmed malignant after 6, 7, 7 and 12 months, respectively.

### Cervical ultrasonography and ^18^F-FDG PET/CT: suspect nodes

Cervical ultrasonography and ^18^F-FDG PET/CT were both suggestive of the presence of cervical lymph node metastases in 26 out of 163 (16%) patients. In 24 out of 26 of these patients FNA was performed, of whom 16 were cytologically confirmed malignant and eight were benign. In two patients it appeared impossible to perform FNA because of the small size and inaccessibility of the lymph nodes. These patients underwent primary oesophagectomy with two-field lymphadenectomy and had no signs of cervical metastases after 12 and 19 months of clinical follow-up, respectively. All false positive nodes (18 out of 26) showed increased ^18^F-FDG uptake and had a short axis diameter ranging between 5 and 10 mm.

### Cervical ultrasonography: suspect nodes

In 12 of 163 (7%) patients, only cervical ultrasonography showed suspect cervical lymph nodes, while ^18^F-FDG PET/CT was negative. FNA showed 10 false positives and two patients had an inconclusive FNA. The patients with inconclusive FNA underwent surgery and had no signs of cervical metastases after 29 and 30 months of clinical follow-up, respectively.

### ^18^F-FDG PET/CT: suspect nodes

In four of 163 (3%) patients only ^18^F-FDG PET/CT was suggestive of cervical lymph node metastases during diagnostic work-up, while cervical ultrasonography was negative. In two patients the suspect nodes were cytologically confirmed malignant (*n* = 1) or benign (*n* = 1) after FNA. In the other two patients FNA was not performed. In one of these patients a CT scan showed enlargement of the cervical lymph node 3 months after initial diagnostic work-up, which was therefore considered malignant. This patient underwent definitive chemoradiotherapy and died a few months after diagnosis of the cervical metastasis. The other patient underwent surgery and showed no cervical metastases during the first 18 months of clinical follow-up.

### Diagnostic performance

The comparison of diagnostic performance between ^18^F-FDG PET/CT and cervical ultrasonography to detect malignant lymph nodes and subsequent subgroup analysis are summarized in Table 2. The sensitivity and specificity of ^18^F-FDG PET/CT was 82% (95% CI 59–94%) and 91% (95% CI 85–95%), respectively. Positive and negative predictive values of ^18^F-FDG PET/CT were 60% (95% CI 41–77%) and 97% (95% CI 23–59%), respectively. The sensitivity of cervical ultrasonography was 73% (95% CI 50–88%), whereas the specificity was 84% (95% CI 77–90%). Positive and negative predictive values of cervical ultrasonography were 42% (95% CI 27–59%) and 95% (95% CI 41–73%), respectively (Table 2). Both sensitivity and specificity were significantly higher for ^18^F-FDG PET/CT scanning compared to cervical ultrasonography. In the various subgroup analyses, ultrasonography did not outperform ^18^F-FDG PET/CT scanning for the detection of cervical lymph node metastasis. Overall, there was no (0% [0/133]) additional value of a cervical ultrasonography to a negative ^18^F-FDG PET/CT scan for the detection of cervical lymph node metastases.

**Table 2:** Comparison of diagnostic parameters of ^18^F-FDG PET/CT and cervical ultrasonography, including subgroup analysis

	^18^F-FDG PET/CT	Cervical ultrasonography	*P* value
Overall			
SE (%) [95% CI]	18/22 (82%) [59–94]	16/22 (73%) [50–88]	0.012
SP (%) [95% CI]	129/141 (91%) [85–95]	119/141 (84%) [77–90]	0.013
PPV (%) [95% CI]	18/30 (60%) [41–77]	16/38 (42%) [27–59]	0.222
NPV (%) [95% CI]	129/133 (97%) [23–59]	119/125 (95%) [41–73]	0.675
Squamous cell carcinomas			
SE (%) [95% CI]	7/9 (78%) [40–97]	6/9 (67%) [30–92]	0.289
SP (%) [95% CI]	52/57 (91%) [81–97]	47/57 (82%) [70–91]	0.063
PPV (%) [95% CI]	7/13 (58%) [36–77]	6/16 (38%) [22–55]	0.477
NPV (%) [95% CI]	52/54 (96%) [88–99]	47/50 (94%) [86–98]	0.929
Level III, IV, V lesions			
SE (%) [95% CI]	17/20 (85%) [62–97]	15/20 (75%) [51–91]	0.008
SP (%) [95% CI]	127/135 (94%) [87–97]	118/135 (87%) [81–92]	0.012
PPV (%) [95% CI]	17/25 (68%) [51–81]	15/32 (47%) [35–60]	0.185
NPV (%) [95% CI]	127/130 (98%) [93–99]	118/122 (96%) [92–98]	0.932
Level II lesions			
SE (%) [95% CI]	2/3 (67%) [9–99]	2/3 (67%) [9–99]	1.000
SP (%) [95% CI]	119/123 (97%) [92–99]	118/123 (96%) [91–99]	1.000
PPV (%) [95% CI]	2/6 (33%) [3–64]	2/7 (29%) [11–56]	0.852
NPV (%) [95% CI]	119/120 (99%) [96–100]	118/119 (99%) [96–100]	0.995

*TP* true positive, *TN* true negative, *FP* false positive, *FN* false negative, *SE* sensitivity, *SP* specificity, *PPV* positive predictive value, *NPV* negative predictive value

## Discussion

In the current cohort study the additional diagnostic value of cervical ultrasonography to ^18^F-FDG PET/CT scanning for the detection of cervical lymph node metastases in patients with newly diagnosed oesophageal cancer was evaluated. Results from the current cohort of patients demonstrated no additional value of cervical ultrasonography to integrated ^18^F-FDG PET/CT scanning.

Several studies suggest that in case of cervical lymph node involvement an aggressive approach with radical oesophagectomy combined with a three-field lymphadenectomy is justified [10]. However, in most Northern American and European countries, presence of cervical lymph node metastases is still considered as systemic disease. Therefore, it is argued that survival will not increase despite removal of these lymph nodes. In that case different palliative therapies are applied for patients with cervical lymph node metastases [11, 12]. Because the presence of cervical lymph node metastases will have a major influence on both prognosis and therapeutic decisions, accurate diagnosis of cervical lymph node metastasis is crucial.

Integrated ^18^F-FDG PET/CT scanning has significantly improved the accuracy of oesophageal cancer staging and frequently influences patient management [13]. Therefore, the use of integrated ^18^F-FDG PET/CT is currently highly recommended in initial oesophageal cancer staging [13]. Owing to the improvement in oesophageal cancer staging by ^18^F-FDG PET/CT scanning, it has been suggested that the additional role of cervical ultrasonography for the detection of cervical lymph node metastasis may be limited [14]. This hypothesis was confirmed in the current cohort of patients in which no additional value of cervical ultrasonography was found to integrated ^18^F-FDG PET/CT scanning for the detection of cervical metastasis. With regard to sensitivity, specificity, PPV and NPV of both diagnostic modalities (Table 2), ^18^F-FDG PET/CT scanning performed better on all these domains compared to cervical ultrasonography. Even though cervical ultrasonography yielded a NPV of 95%, this did not result in an additional value over ^18^F-FDG PET/CT which yielded an even higher NPV of 97%. Also, no subgroup in which ultrasonography outperformed ^18^F-FDG PET/CT scanning for the detection of cervical metastasis could be identified. However, suspected cervical lesions detected with ^18^F-FDG PET/CT still require cytopathological confirmation by FNA because of possible false positives (encountered in 12 of 163 [7%] patients in the current study).

Our results are consistent with two articles in the literature that reported no additional value of cervical ultrasonography to integrated ^18^F-FDG PET/CT scanning (or standalone PET combined with CT) for detecting cervical lymph node metastases (*n* = 170 and *n* = 136, respectively) [14, 15]. One study found an additional value of 4% (3/74) of cervical ultrasonography over standalone PET and CT imaging (*n* = 109) [16]. Methodological differences between the various studies could explain some of the discrepancies in reported added value of cervical ultrasonography. First, two studies only assessed standalone PET and CT [15, 16]. This may have led to an underestimation of the diagnostic value of PET/CT, as standalone PET and CT may result in lower diagnostic accuracies compared to integrated ^18^F-FDG PET/CT scanning. To this regard, the only study so far that used integrated ^18^F-FDG PET/CT scanning demonstrated no additional value of cervical ultrasonography for the detection of cervical lymph node metastases [14]. The current study was able to confirm these results in an independent set of patients, which suggests external generalizability to other patient populations. Second, criteria for positive cervical lymph nodes on both ^18^F-FDG PET/CT and cervical ultrasonography were heterogeneous throughout the different studies, and were often operator-dependent [14–16]. However, the results of the current study and those present in the current literature represent cervical lymph node staging in daily practice [14–16].

In the current cohort study, the incidence of cervical lymph node metastases was 14% (22/163). This rate is high compared to the other studies assessing the additional value of cervical ultrasonography, which reported incidences of cervical lymph node metastases ranging between 3% and 9% [14–16]. This difference can be explained by the fact that the current study included a relatively high number of proximal (13%) and squamous cell tumours (40%) compared to the other studies. It is well known that these types of tumours, at these locations, have a higher incidence of cervical lymph node metastases [17]. Even higher incidences of 27–31% have been described in case extensive pathological examination is performed after three-field lymph node dissection [18, 19].

Potential limitations apply to this study. First, in our cohort study the image analysts were not blinded to the results of earlier conducted investigations, which may have influenced the interpretation of the different tests. Second, cervical lymph node metastasis may have been missed by both imaging modalities, as no pathological evaluation was available for patients with a negative test. These patients were evaluated by clinical follow-up, which is a potentially less reliable reference test. To this regard, differential verification bias was of concern in the current study because different reference standards were used for the detection of cervical lymph nodes [20]. Third, the time interval (e.g. 6 or 12 months) in which positive findings at follow-up are regarded as false negatives influences the diagnostic values of the different modalities. Changing the time interval of the current study from 12 to 6 months, for example, would have resulted in an overestimation of the reported sensitivities of both modalities. However, in the current study changing the time interval to 6 months would not have influenced the added value of cervical ultrasonography over ^18^F-FDG PET/CT.

In conclusion, this study demonstrates that cervical ultrasonography has no additional diagnostic value over a negative ^18^F-FDG PET/CT for the detection of cervical lymph node metastases in patients with newly diagnosed oesophageal cancer. Especially since the introduction of integrated ^18^F-FDG PET/CT systems, cervical ultrasonography during standard diagnostic work-up of oesophageal cancer patients may be considered unnecessary. Suspected cervical lesions on ^18^F-FDG PET/CT still require cytopathological confirmation by FNA because of possible false positive results.
